# An Evaluation of Phase Analysis to Interpret Atrial Activation Patterns during Persistent Atrial Fibrillation for Targeted Ablation

**DOI:** 10.3390/jcm11195807

**Published:** 2022-09-30

**Authors:** Seungyup Lee, Celeen M. Khrestian, Jayakumar Sahadevan, Albert L. Waldo

**Affiliations:** 1 Departments of Medicine, Case Western Reserve University, Cleveland, OH 44106, USA; 2 Department of Biomedical Engineering, Case Western Reserve University, Cleveland, OH 44106, USA

**Keywords:** atrial fibrillation mechanisms, persistent atrial fibrillation, mapping, phase analysis, rotor

## Abstract

Background: Phase analysis has been used to identify and localize atrial fibrillation (AF) sources for targeted ablation. We previously demonstrated that repetitive wannabe reentry (incomplete reentry) often generated an apparent stable rotor using phase analysis. The misinterpretation caused by phase analysis using atrial electrograms (AEGs) may result from detecting inaccurate time points at phase inversion (π to −π) in the instantaneous phase waveform converted from AEG. The purpose of this study was to evaluate the accuracy of phase analysis to detect atrial activations recorded from the high-density mapping of AF in patients with persistent and long-standing persistent (LSP) AF. Methods and Results: During open heart surgery, we recorded activation from both atria simultaneously using 512 electrodes in 7 patients with persistent and LSP AF. The phase analysis was compared to manual measurements during 4 s of data. For the accuracy of activation sequence maps, a successful recording site was defined as having ≤4 mismatched activation times during the 4 s. In all AF episodes, the accuracy of the phase analysis was only 82% of the total number of activation times due to either activation time differences (14.7%), under-sensing (2.7%), or over-sensing (0.6%). Only 67.9% of the total recording sites met the requirement of a successful recording site by phase analysis. In unsuccessful recording sites, AEG characteristics were relatively irregular cycle length (CL), complex AEG, and double potential AEG. Conclusion: The phase analysis was less accurate in recording sites with a relatively irregular CL, complex AEG, or double potential AEG. As a result, phase analysis may lead to the misinterpretation of atrial activation patterns during AF. A visual review of the original AEG is needed to confirm the detected AF sources of phase analysis before performing targeted ablation.

## 1. Introduction

Many atrial mapping studies using phase analysis in patients with atrial fibrillation (AF) have described the presence of rotor or spiral reentry suggestive of or thought to act as sources maintaining AF [1,2,3]. However, recent mapping studies conducted in patients with persistent AF have shown that phase analysis produces a different mechanistic result than classical activation sequence analysis [4,5,6]. Our recent study in patients with persistent and long-standing persistent (LSP) AF demonstrated that repetitive wannabe reentry (incomplete reentry) generated by a focal activation or a passive wavefront identified by activation sequence mapping often produced a stable rotor identified by phase mapping separate from the focal activation or a passive wavefront [7]. Additionally, the study showed that high-density mapping using phase analysis could not distinguish between a true rotor and a wannabe reentry. These limits of phase analysis conducted using atrial electrograms (AEGs) may result from inaccurate time points at phase inversion (π to −π) in the instantaneous phase waveform converted from AEG. In addition, AEG characteristics may have an effect on the accuracy of phase analysis in detecting a local atrial activation time. The purpose of this study was to evaluate the accuracy of phase analysis to detect atrial activations during the high-density mapping of AF in patients with persistent and LSP AF. The time points at phase inversion in the instantaneous phase waveform were used as local atrial activation times. Using the manual measurement of activation times as the gold standard, we assessed the accuracy of phase analysis to detect activation times and mean CL. Furthermore, we investigated AEG characteristics associated with the inaccurate detection of atrial activation times when using the phase analysis.

## 2. Materials and Methods

The research protocol was approved by the Institutional Review Board at University Hospitals Cleveland Medical Center. Seven patients with persistent and LSP AF (1 month–9 years duration) were studied during open heart surgery (Table 1). After the heart was exposed using standard surgical procedures during open heart surgery, atrial epicardial mapping studies were performed in patients with persistent and LSP AF [8,9]. Three electrode arrays with a total of 512 electrodes (left atrium—256 electrodes; right atrium—160 electrodes; Bachmman’s bundle—96 electrodes) were placed on the atrial epicardial surface of both atria for simultaneous recording, as previously described [8]. The interelectrode distance between bipolar electrode pairs was 1.5 mm, and the distance between the center of each bipolar pair and its neighbors varied from 5.2–7.0 mm. AEGs from 512 electrodes (256 bipolar pairs) along with ECG lead II were simultaneously recorded for 1–5 min during persistent AF. All AEGs were gained at 1000, sampled at 1024 Hz, and digitized at 24 bits with an Active Two system (BioSemi, Amsterdam, The Netherlands). Data were transferred in real-time and stored on a laptop computer for further analysis (Cardiac Electrophysiology Analysis System [CEPAS], Madry Technologies, Sydney, Australia).

(a) Manual Measurement of Atrial Activation Times

During each of 7 episodes of persistent and LSP AF, all activation times of bipolar AEGs recorded over 4 s were carefully measured by two investigators who were experienced in the mapping of AF. The following was the method of selection of bipolar activation times used in our study, as previously described [8]. The moment of activation at each site was taken as (1) for sites in a predominantly monophasic AEGs, the peak of the rapid deflection, (2) for sites in a predominantly biphasic AEGs, the time when the first rapid deflection crossed the baseline, or (3) for sites in which polyphasic electrograms (AEGs with two or more deflections) were recorded, activation was assigned to the major deflection (highest amplitude or fastest downstroke). If there was a double potential (2 discrete deflections during 1 atrial complex in the AEGs), the activation was assigned to the highest-amplitude peak or unipolar QS morphology. Our bipolar pairs were closely spaced (1.5 mm), so that continuous electrical activity (fractionation) was rarely seen, consistent with published data [10]. Activation from neighboring sites was also used to aid in determining the activation of complex electrograms recorded during high-density mapping. Recording sites were excluded from analysis if a site had either no atrial electrograms recorded or only 1 unipolar electrogram recorded. The same 4-s segment was then subjected to the phase analysis (see following).

(b) Phase Analysis

**Figure 1:**
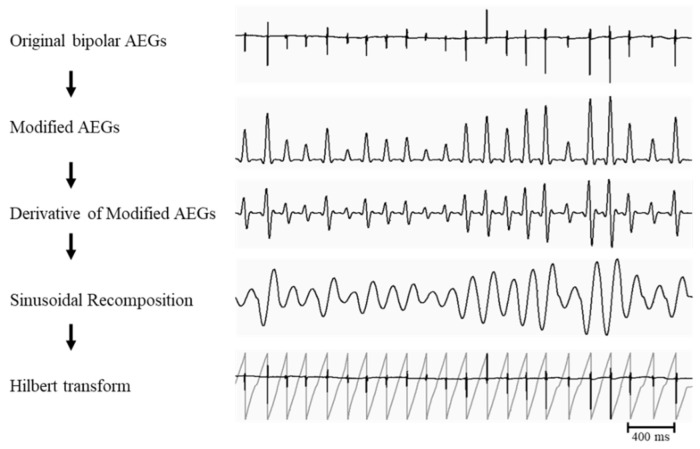
Flow chart of the phase analysis and the signal processing procedures that were applied to the original bipolar AEGs. AEGs, atrial electrograms.

Phase analysis of bipolar AEGs using phase mapping was performed using a customized algorithm incorporated into the analysis software (CEPAS, Madry Technologies, Sydney, Australia). The phase analysis for bipolar AEGs can be summarized in the following steps (Figure 1): (1) modified AEGs were constructed using a previously described filtering method [11]. The peaks of the modified AEGs produced only smooth local maximum amplitudes that were related to each local activation regardless of polarity and morphology of the bipolar AEGs; (2) The estimated mean cycle length (CL) was calculated by dominant frequency (DF) analysis of the modified AEGs. The estimated mean CL was calculated as 1000/DF peak (milliseconds); (3) for the sinusoidal-recomposition method [12], each modified AEG was transformed into a derivative waveform in order to produce a local maximum negative slope at each peak (local activation times) of modified AEGs; (4) the sinusoidal-recomposition was performed using the estimated mean CL previously calculated by DF analysis. The amplitude of the sinusoidal waveform is proportional to the negative slope of the derivative waveform; (5) Hilbert transform was applied for phase mapping; (6) using zero-crossing detection, local atrial activation times were determined at the time points at phase inversion (π to −π) in the instantaneous phase waveform.

(c) Evaluation of the phase analysis

All detected activation times and mean CL of the phase analysis were compared with the manual measurement using linear regression. The evaluations of comparisons were assessed by Pearson’s correlation coefficient (CC). Additionally, for measuring the accuracy of the phase analysis, the manually detected activation times at each recording site were compared with the phase analysis to identify mismatched activation times. A mismatched activation time was defined as (1) activation time differences > 10 ms, (2) under-sensing (fail to detect an activation time by phase analysis within 75 ms of a manually marked activation time), or (3) over-sensing (fail to match an activation time by manual measurement within 75 ms of activation time by phase analysis). A successful recording site was defined as having ≤4 mismatched activation times between phase analysis and manual measurements. To further investigate the performance of the phase analysis, the AEG characteristics of gold standard measurement for mean CL and standard deviation (SD) were compared between successful recording sites and unsuccessful recording sites. Additionally, the organization index (OI) calculated by DF analysis was used as the indicator of organized AEG morphology [11]. The OI was calculated as the ratio of the area of the dominant peak ±0.5 Hz and its harmonics to the total area (4–50 Hz) of the magnitude spectrum. The greater the OI, the higher degree of organization of AEG. The OI was compared between successful and unsuccessful recording sites. Since the phase analysis could be limited by CL variability, the degree of CL regularity over time was assessed using SD. Specific cutoffs (SD < 10, 15, 20, 30 ms) of CL regularity at each site were used as a variable parameter when evaluating the accuracy of the phase analysis. The performance of the analysis at each cutoff degree of CL regularity was evaluated for accuracy. Additionally, specific AEG characteristics were examined on unsuccessful recording sites.

## 3. Results

### 3.1. The Phase Analysis Compared to Manual Measurement

**Figure 2:**
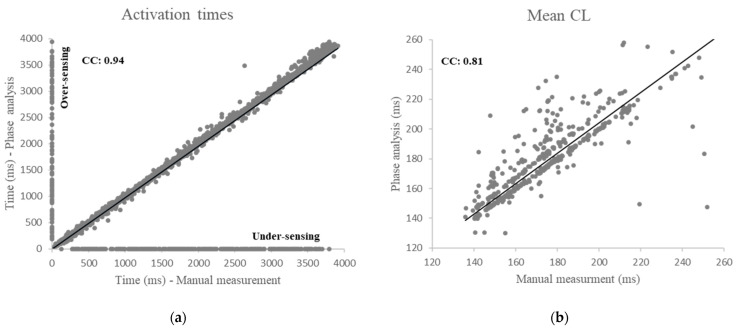
Intraclass correlation of the phase analysis compared to manual measurement. (**a**): Correlation between activation times detected by the phase analysis and the manually measured activation times. (**b**): Correlation between the mean CL detected by the phase analysis and the manually measured mean CL. CL, cycle length.

All activation times and the mean CL at each bipolar AEGs determined using the phase analysis were compared to manually measured activation times and mean CL during AF in patients with persistent and LSP AF. A very strong correlation (CC: 0.94) was found between manually measured activation times and activation times detected by the phase analysis (Figure 2a). A strong correlation (CC: 0.81) was also found between manually measured mean CL and mean CL detected by the phase analysis (Figure 2b). However, the accuracy of the phase analysis was only 82% of total activation times due to either activation time differences (>10 ms) between the manual measurement and the phase analysis (14.7%), under-sensing (2.7%, dots in the *x*-axis, Figure 2a), or over-sensing (0.6%, dots in the *y*-axis, Figure 2a). Additionally, the accuracy of the phase analysis for a successful recording site (mismatched activation ≤ 4 over 4 s) was only 67.9% of total recording sites.

### 3.2. Accuracy of the Phase Analysis Dependent on AEG Characteristics

**Figure 3:**
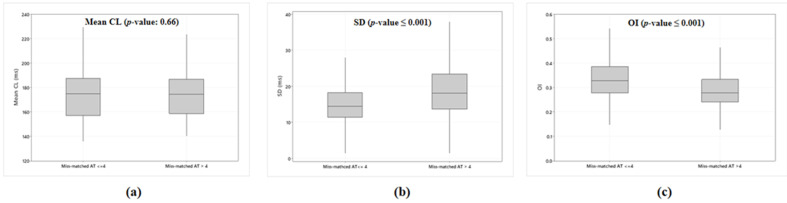
Box plots illustrating the difference of (**a**) mean CL, (**b**) SD, and (**c**) OI between successful recording sites (mismatched activation time ≤ 4) and unsuccessful recording sites (mismatched activation time > 4). Each box plot illustrates each range obtained from the manual measurement of both mean CL, SD, and OI. AT, activation time; CL, cycle length; OI, organization index; SD, standard deviation.

To understand further the lack of accuracy of the phase analysis in determining the true activation times, the AEG characteristics of gold standard measurement for both mean CL and SD, and OI, were compared between successful recording sites (mismatched activation ≤ 4 by phase analysis) and unsuccessful recording sites (mismatched activation > 4 by phase analysis). There was no difference in the manually measured mean CL when comparing successful to unsuccessful recording sites (*p* = 0.66, Figure 3a). However, SD of successful recording sites was significantly less than those of unsuccessful recording sites (*p* < 0.001, Figure 3b). Additionally, OI of successful recording sites was significantly higher than unsuccessful recording sites (*p* < 0.001, Figure 3c).

The phase analysis could be limited by CL variability, therefore, the degree of CL regularity over time was assessed using manually measured SD. Table 2 shows the accuracy of the phase analysis with varying degrees of CL regularity as determined by different SD cutoffs from the most regular (SD < 10 ms) to the least regular CL (SD ≥ 10 but <30 ms) of AEGs. The accuracy of the phase analysis was increased from 82% to 83%, 88%, 94%, or 98% when decreasing the SD cutoffs from 30 ms to 20 ms to 15 ms to 10 ms, respectively. The accuracy at the relatively regular CL (SD ≤ 15 ms) using the phase analysis was over 90%.

**Figure 4:**
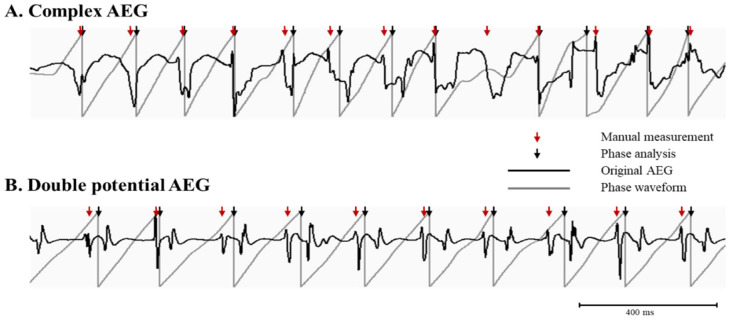
A representative example of inaccurate activation times detected by phase analysis. Bipolar AEGs (black color) with their phase (gray color) are shown in AEG characteristics having complex AEGs (panel **A**) and double potential AEG (panel **B**). Arrows indicate activation times from manual measurement (red color) and phase analysis (black color).

Table 3 shows the AEG characteristics for unsuccessful recording sites when using phase analysis. The common AEG characteristics on unsuccessful recording sites were complex or double potential AEGs. Figure 4 showed representative examples of inaccurate activation times detected by phase analysis on complex AEG (Figure 4A) and double potential AEG (Figure 4B). Both original AEG (black color) and phase waveform (gray color) were shown with activation times measured manually (red arrow). The time points at phase inversion in the phase waveform were detected activation times by phase analysis. Multiple deflections on AEGs resulted in the distortion of phase waveform, which produced inaccurate phase inversion. For example, in the double potential AEGs with stable CL (Figure 4B), the inaccurate phase inversion of phase waveform consistently occurred.

## 4. Discussion

### 4.1. Major Finding

All AF activation times detected by the phase analysis were compared to manually measured activation times in patients with persistent and LSP AF. This study demonstrated that the accuracy of phase analysis was 82% of the total number of activation times compared to manual measurement recorded during AF. Only 67.9% of the total recording sites detected by the phase analysis met the requirement of a successful recording site, which was due to either activation time differences (>10 ms), under-sensing, or over-sensing when compared to manual measurement. Those mismatched activation times resulted from AEG characteristics having relatively irregular CL, less organized AEG, complex AEG, and double potential AEGs. Although the phase analysis is most likely accurate in AEGs with stable CL, the inaccurate phase inversion of phase waveform consistently occurred in the double potential AEGs with stable CL.

### 4.2. Targeted Ablation in Persistent and LSP AF Using the Signal Processing Algorithms

Several signal processing methods have been applied over the past 20 years to characterize AEGs and analyze repetitive activation patterns (focal or rotational) of AF for targeted ablation [1,2,11,12,13,14,15,16,17,18,19,20,21,22,23,24]. Two major and important analysis approaches are (1) characterizing AEGs to identify the sites of AF sources (e.g., complex fractionated atrial electrograms (CFAEs), DF, low voltage of bipolar AEGs), and (2) the identification of activation patterns that are distinctive for AF sources (e.g., repetitive focal or rotational activation). For the approach using AEG characteristics, the analysis algorithms have used atrial rate, CL regularity, and morphology characteristics in both the time and frequency domains. However, the approach using AEG characteristics alone cannot reliably distinguish between active AF sources and “irrelevant” areas (e.g., fibrillatory conduction) without contemporaneously identifying activation sequences [10,25,26,27]. Additionally, the results are often contaminated due to poor AEGs quality, inadequate bipolar interelectrode distance, and far-field effects (remote electrical activity) [27,28,29,30,31]. Therefore, recent clinical trials showed that the rate of freedom from AF in patients who underwent pulmonary vein isolation (PVI) plus targeted ablation of specific AEG characteristics, e.g., CFAEs, DF, low-voltage, and MRI-guided fibrosis, is not superior to those who underwent PVI alone [17,32,33,34]. For the approach using the identification of activation patterns, both activation sequence and phase analyses have been used in the time domain [11,22,23,24]. However, the approach using the identification of activation patterns distinctive for an AF source is extremely dependent on high interelectrode spatial resolution for accuracy, and understanding activation sequences precisely in real-time is challenging [27,29,30,31].

Phase analysis to identify AF sources using the instantaneous phase of electrograms has recently been proposed [12,35,36]. Several investigators have used phase analysis for targeted ablation in patients with persistent and LSP AF with favorable outcomes [1,2,3]. However, recent mapping studies have demonstrated that phase analysis could overestimate the number of rotational activation patterns during AF [4,5,6,7]. Most previous studies did not consider why phase analysis produces false positive rotational activation patterns compared to classical activation sequence analysis when using the same data. In this study, we investigated the accuracy of phase analysis to detect atrial activation times of AF. Our study showed mismatched activation times due to either activation time difference (>10 ms), over-sensing, or under-sensing. Although our findings of the correlation coefficients of both activation times and mean CL between the phase analysis and manual measurement were similar to the finding of others [35,36], the phase analysis was inaccurate due to either activation time differences, under-sensing, or over-sensing. Additionally, we demonstrated that the phase analysis is inaccurate on AF AEGs characteristics having relatively irregular CL, less organized AEGs, and double potentials. Therefore, those mismatched activation times of the phase analysis may lead to false identification of AF sources and unnecessary ablations.

### 4.3. Implications

In an attempt to identify and localize AF sources, many analysis algorithms have been proposed and applied for detecting activation times in real-time during AF. However, clinical studies showed that AF AEGs might exhibit variable large amplitude or morphology artifacts due to electrical far-field noise that obscures true local activation. Therefore, precisely determining activation times in real-time continues to be a challenge. Recent AF mapping studies demonstrated that one mechanism of AF might be due to one or more sources characterized by repetitive activation patterns manifesting rapid and regular CL [37]. Thus, the AF analysis algorithm may focus on identifying and localizing regular activity within the overall irregularity during AF. We determined successful recording sites using phase analysis as having mismatched activation ≤ 4 during 4 s. The mismatched activation determination was based on three criteria, activation time difference > 10 ms, under-sensing, or over-sensing. Since the activation sequence map normally has a 10-ms isochronal scale, a site with a false activation time (>10 ms of manual measurement) may provide an error in interpretation of the activation sequence map. Only 67.9% of total recording sites met the requirement of a successful recording site by phase analysis. Although the accuracy of phase analysis at the relatively regular CL was over 90%, the inaccurate phase inversion of phase waveform consistently occurred in the double potential AEGs having stable CL. In unsuccessful recording sites, AEG characteristics had relatively irregular CL, less organized AEG, complex AEG, and double potential AEG. In particular, since recorded AEG from areas in dilated cardiomyopathy, i.e., scar, manifests more complex AEGs [38,39], the phase analysis may be less accurate in AF patients with structural abnormalities in the atrial wall. Therefore, the detected AF sources of phase analysis should undergo a review of the original AEGs to confirm the analysis before performing targeted ablation. This review is expected to take less than 5 min to display the relevant sites to verify a complete rotation by a cardiac electrophysiologist.

### 4.4. Limitations

First, the ventricular subtraction was not performed before the phase analysis because bipolar AEGs have a low ventricular far-field incident. Additionally, applying the ventricular subtraction in all recording sites may produce large distortions of original AEGs since each recording site of high-density mapping has variable ventricular far-field effects based on electrode location or unipolar subtraction. After ventricular subtraction, the result of the phase analysis may be different. Second, the phase analysis was only performed on 4 s of data. However, since a minimum duration (>2 s) is needed for DF analysis in calculating an estimated mean CL for the phase analysis, the duration of the analysis is not a factor in the accuracy of the phase analysis. Third, the definition of a successful recording site may change depending on the analysis window chosen, i.e., mismatched activation time ≤ *N* during *N* second. Finally, the fact that our study population is small and all patients had valvular heart disease may make this patient population unique when applying the phase analysis during AF.

## 5. Conclusions

The phase analysis was tested and validated against manual measurement from recorded AEGs in patients with persistent and LSP AF. The phase analysis was inaccurate in recording sites with relatively irregular CL, complex AEG, or double potential AEG. As a result, phase analysis could provide a misinterpretation of atrial activation patterns during AF. An additional analysis algorithm that qualifies recording sites with regular CL and organized AEGs may improve the accuracy of identifying repetitive activation patterns (AF sources) characterized by rapid and regular CL in real-time. Alternatively, for the AF sources detected in the phase analysis, a visual review of the original AEG is needed to confirm the analysis before performing targeted ablation.

## Figures and Tables

**Table 1:** Patient Characteristics.

Patient No.	Age	Gender	AF Duration	Valvular Disease	CAD	Heart Failure
1	60	M	>1 year	MR	−	+
2	57	M	>1 year	MS	−	+
3	67	M	1 month	MR	−	+
4	70	M	9 years	AS, TR	−	+
5	70	F	8.5 years	AS	−	+
6	80	F	2.5 years	TR	−	+
7	63	M	>1 year	MR, TR	+	+

AF, atrial fibrillation; AS, aortic stenosis; CAD, coronary artery disease; MS, mitral stenosis; MR, mitral regurgitation; TR, tricuspid regurgitation; +, present; and −, absent.

**Table 2:** The accuracy of phase analysis at different SD cutoff compared to manual measurement.

SD Cutoff	Accuracy of Phase Analysis
SD < 10 ms	98%
SD < 15 ms	94%
SD < 20 ms	88%
SD < 30 ms	83%
Overall	82%

**Table 3:** AEG Characteristics Resulting in Unsuccessful Recording Sites by Phase Analysis.

AEG Characteristics				
Complex AEG	Double Potential	Ventricular Complex	Variable Amplitude	Others
61%	16%	12%	8%	3%

## Data Availability

All data supporting the findings of this study are available within the article.
